# Two Remarkable Cases of Abstinence

**Published:** 1851-10

**Authors:** 


*Two Remarkable Cases of Abstinence.* By Dr. Taylor.—The *first* of these cases occurred in a man, æt. 50, who, after commercial disasters, began to manifest some symptoms of insanity, one of which was obstinate refusal of food. For *ten* days and nights he entirely abstained from food or drink, but then by stratagem was induced to take a hearty meal. This was followed by another period of abstinence for fourteen days and nights, during which period he passed stools once and urine three times. He slept well, walked about the room occasionally, and suffered neither from fever nor exhaustion; on the 15th day he took a little water, and in future took a little of this as well as a little milk—not above a gill per diem, sometimes refusing it for days together. This state of things continued until his death, he having lived in a state of almost complete abstinence for about 100 days, a demulcent injection having been administered on rare occasions. Independently of the emaciation, he presented no symptom of disease. He passed urine every ten days, and a slight stool every fifteen days.

Mr. ——, the subject of the second case, æt. 26, after manifesting other symptoms of mental derangement, lived during one year, eighteen weeks, and sixteen days in an almost complete state of starvation, taking nothing during the last fifty-one days but a little water occasionally. He had no fæcal evacuations for seventy-two days prior to death, but passed small quantities of urine every three or four days.—*Amer. Jour. Med. Sc.*

